# Dwarfism associated with wandering splenomegaly

**DOI:** 10.1093/jscr/rjab558

**Published:** 2022-01-22

**Authors:** Andy Petroianu, Kelly Renata Sabino

## Abstract

Dwarfism associated with splenomegaly during teenage years is known. The purpose of this report was to present the first case of dwarfism associated with wandering splenomegaly. A 14-year-old boy presented a wandering splenomegaly and torsion of the splenic pedicle associated with a retarded growth and sexual underdevelopment characterized by lack of sexual maturity and absence of secondary sexual physical appearance. The patient was submitted to detorsion of the spleen, and splenopexy. After the surgery, the patient grew up, and his sexual characteristics developed to normal. The size of the spleen reduced from the 22 × 16 × 13 cm to 14 × 12 × 10 cm after the surgical procedure. This is the first report of dwarfism associated with wandering splenomegaly, which was successfully treated without partial or total splenectomy. After releasing the venous blood flow, the spleen reduced its dimension and the patient grew up to the expected family size.

## INTRODUCTION

Dwarfism associated with splenomegaly during childhood and teenage years has been described in the literature since the 1950s [1–3]. Total or partial splenectomy before consolidation of the bone epiphyses is recommended in order to remove possible splenic impairment to body growth and sexual development [2–4]. This treatment has been successfully performed in many patients, but with subsequent asplenia, which is related to serious adverse effects. Metabolic disorders, infections and death due to overwhelming sepsis, as well as pulmonary embolism, have often been reported after total splenectomy [3, 5].

Splenoptosis or wandering spleen is a rare clinical condition defined as an ectopic spleen moved from its normal anatomical position in the left hypochondrium caused by the congenital absence or malformations of perisplenic ligaments [6–8]. Complications, including the splenic pedicle torsion, which is responsible for splenomegaly, are usually found in wandering spleens [9–12]. Other disorders associated with this condition should be also considered and investigated.

The nonspecific clinical symptoms, consisting mostly of abdominal pain, make the definition of this diagnosis challenging. Computed tomography (CT) is the best imaging exam to make this diagnosis, but ultrasound may be useful as well. The first-line treatment is elective laparoscopic spleen detorsion and splenopexy [9–13].

The purpose of this communication is to present the first published case of dwarfism associated with wandering splenomegaly, which was successfully treated by splenic detorsion and splenopexy.

## CASE REPORT

**Figure 1:**
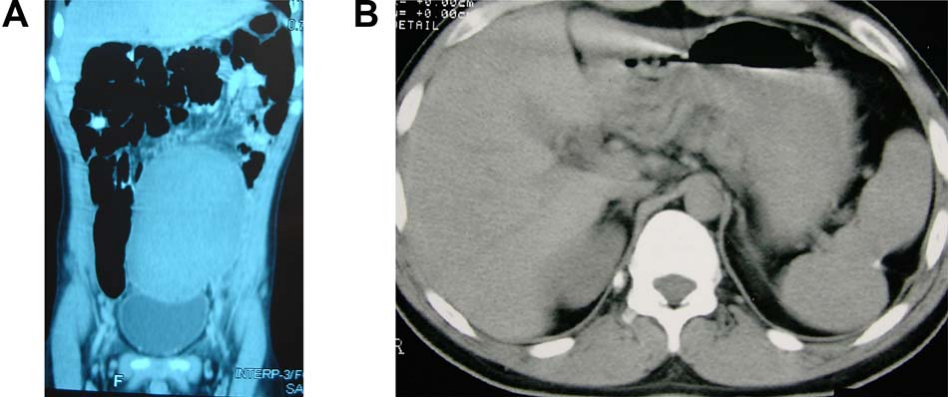
Wandering and twisted splenomegaly. (**A**) CT scan of the abdomen showing a displaced ectopic spleen in the left flank and pelvic region, characteristic of a wandering spleen. Observe the absence of the spleen in the left hypochondrium. (**B**) The spleen after detorsion and splenopexy in the left hypochondrium. Observe the reduction of the spleen to a normal size four years after the splenopexy.

A 14-year-old boy was referred to our hospital due to a long-term history of lower abdominal pain associated with dysuria, tenesmus and constipation associated with a visible tender pelvic mass. He also exhibited a retarded growth (152 cm in height) and sexual underdevelopment characterized by lack of sexual maturity and absence of secondary sexual physical appearance with no facial, pubic and axillary hair growth. Laboratory exams indicated a reduced platelet count of 114 × 10^3^/μl, a white blood cell count of 6.2 × 10^3^/μl and a hemoglobin level of 11.2 g/dl. An axial CT showed the absence of the spleen in the left subphrenic space and an enlarged spleen, measuring 22 × 16 × 13 cm, within the pelvic region (Fig. 1A).

Laparotomy was recommended, and an enlarged, twisted spleen without perisplenic ligaments was found in the lower abdomen. After the detorsion of the spleen and its vascular pedicle, the spleen was located in its proper left subphrenic site and a splenopexy was performed with four stitches to the diaphragm and left upper peritoneum, using a 2–0 polyglactin 910 thread. The stitches were placed on the superior (one) and lateral (three) borders of the spleen, by including about 1 cm of parenchyma to avoid the splenic tear during the stitches binding. The bleeding was prevented by the attached peritoneum to the spleen.

The patient had an uneventful recovery and was discharged from the hospital on the second postoperative day. The most significant body growth occurred during the first 9 months after the surgery, and at 18 years of age, he had grown to 183 cm in height, similar to his family size. Together with the body height, all sexual characteristics were improved to normal aspects short time after the splenopexy. All laboratory exams had returned to normal, including a platelet count of 280 × 10^3^/μl, a white blood cell count of 6.4 × 10^3^/μl and a hemoglobin level of 14.9 g/dl. A new CT image demonstrated an almost normal spleen (14 × 12 × 10 cm) with no splenic or abdominal disorder (Fig. 1B). After four years, the patient is still in good health with a normal life, with no complaint related to the surgical procedure.

## DISCUSSION

The ectopic spleen is a rare condition, which is usually asymptomatic, but it may be responsible for chronic abdominal pain with gastrointestinal and urinary symptoms [9–15]. Patients with torsion of the wandering spleen may not only have acute abdomen or chronic and intermittent symptoms, but also splenomegaly and splenic infarction as a result of vascular congestion [7, 8, 10, 14].

The treatment should consider the risk of post-splenectomy overwhelming sepsis, mainly in pediatric, elderly and chronic illness patients, which is around 5% [3, 5]. Laparoscopic spleen detorsion and splenopexy seems to be the best treatment, mainly to avoid the complications of the asplenic condition [9–12]. A relevant aspect of this communication is the reduction of a congestive splenomegaly to a normal size spleen after detorsion of the splenic vessels and splenopexy to the left diaphragm.

Although it was first described 70 years ago, the dwarfism and delayed puberty associated with splenomegaly in its early ages has not been studied in proportion to its importance [1–4]. Very few papers have been published on this subject, most of which have been case reports. No investigation of this disorder can be found in the literature. The spleen is still the least studied organ and no knowledge is available to link the spleen with the body and sexual development. Splenomegaly seems to inhibit the different phases of human growth, most likely by means of endocrine mediators. Growth hormones and other mediators related to the body and sexual development may be impaired inside the spleen when the splenic blood flow is reduced by a venous obstruction or due to metabolic or hematological disorders. Although it is not an endocrine organ, an enlarged spleen may produce mediators that can interfere with the growth hormone and reduce its efficacy.

Even without knowing the mechanism of the dwarfism associated with splenomegaly, total or partial splenectomy performed before the epiphyseal consolidation is generally followed by body and sexual development that runs in line with family characteristics [1–4]. The same good results are achieved if a congestive splenomegaly is treated by releasing the venous blood flow from its obstruction. In this case report, the splenic detorsion improved the venous blood flow and the congestive splenomegaly was reduced to a normal splenic size [9–13, 15] (Fig. 1). Thus, this report demonstrates that dwarfism due to a congestive splenomegaly should only be treated aiming at an appropriate venous blood flow.

## CONCLUSIONS

This is the first report of dwarfism associated with wandering splenomegaly, which was for the first time successfully treated without partial or total splenectomy. After releasing the venous blood flow, the spleen reduced its dimension to normal and the patient grew up to the expected family size.

## COMPETING AND CONFLICTING INTERESTS

The authors declare they have no competing or conflicting interests.

## FUNDING

None.

## ETHICAL APPROVAL

The patient signed an informed consent form. Ethical approval was not required, considering this paper reports the treatment performed on a patient with no research intention.

## CONSENT

Written informed consent was obtained from the patient for publication of this case report and accompanying images. A copy of the written consent is available upon request.

## AUTHOR CONTRIBUTIONS

A.P. worked on conceptualization, data curation, formal analysis, investigation, methodology and performed the surgical procedure. He also assisted the patient and contributed in writing, reviewing and editing the original draft. He was also responsible for all information and submitting of the article for publication. K.R.S. worked on data curation, formal analysis and investigation and also assisted the patient and contributed in writing of original draft.
